# Progress in Surgery

**Published:** 1897-10-30

**Authors:** 


### Progress in Surgery.

### LIVER, GALL-BLADDER, AND DUCTS.

(*Continued from page* 66.)

### Gall-bladder and Ducts.

This subject has been traversed in the recent lectures at the Royal College of Surgeons by Mr. Mayo Robson,[11] which lectures may well form the groundwork for these notes, and other literature can be added afterwards.

**Anatomical Points.**—The middle-third of the common duct and the upper two-thirds of the cystic duct are the only convenient positions for choledochotomy, because the portal vein is on the outer side and front of the common duct at its upper third, and also the termination of the cystic duct; moreover, a branch of the pancreatico-duodenal artery and other vessels cross over the common duct. There are glands normally in the free border of the lesser omentum which may be mistaken for calculi. The large right lumbar pouch of peritoneum must be remembered for drainage purposes.

**Chronic Catarrh of the Gall-bladder** may be treated as we do catarrh of the urinary bladder—first, by medicine and general remedies; secondly, if necessary, by securing physiological rest by drainage.

**Cholecystitis or Suppurative Catarrh** of the gall-bladder has various origins, according to the micro-organisms present. Bile is normally sterile, but probably invariably infected in disease of the gall-bladder or ducts, by such organisms as the bacillus coli communis in phlegmonous cholecystitis, the typhoid bacillus after enterica, and in one case the pneumococcus. It is a striking fact that this typhoid bacillus is usually present in the gall-bladder after death from typhoid. (*a*) Simple suppuration (empyema) is most frequently associated with gall-stones. The tumour, at first freely moveable, becomes fixed later owing to the spread of the inflammation to the peritoneum. An abscess forms which may discharge at the umbilicus, costal margin, hypogastrium, cæcum, neighbouring organs, and elsewhere. The treatment of empyema of the gall-bladder is cholecystostomy, with removal of any cause which may be present, or cholecystectomy if the gall-bladder be rotten. (*b*) Acute phlegmonous suppuration of the gall-bladder is very fatal, and may end in general peritonitis, or gangrene from thrombosis of the nutrient arteries. It may occur with or without gall-stones; for example, in sloughing inflammation of the bowels, puerperal fever, and pyæmia. It has to be diagnosed from appendicitis. The treatment is similar to that of the foregoing, only the necessity is more urgent.

**Infective or Suppurative Inflammation of the Bile-ducts** (suppurative cholangitis). The chief causes are cholelithiasis, cancer, hydatid disease, and suppurative diseases in the portal area. Treatment: Cholecystotomy and drainage, drainage of any liver abscess, mercurial purges, and intestinal antiseptics.

**Ulceration of the Gall-bladder and Bile-ducts** occurs in cholelithiasis, cancer, and typhoid. It usually leads to localised plastic peritonitis, sometimes to general suppurative peritonitis, and perhaps cancer. Occasionally serious hæmorrhage occurs.

**Stricture** is often the result of gall-stones or ulceration. It will give rise to jaundice on the one hand, or enlargement of the gall-bladder on the other. The former is caused by stricture of the common duct, when short circuiting is necessary; the latter by stricture of the cystic duct, which requires extirpation or drainage of the gall-bladder for its treatment.

**Perforation of Gall bladder or Bile-duct.**—If the bile be from a healthy gall-bladder, and surgical relief be early, recovery may ensue, as in Thiersch's case, in which 40 pints of healthy bile were in the peritoneal cavity, and in Hans Kehr's case, in which a gunshot wound of the gall-bladder was sewn up with recovery. The prognosis is much more serious if the bile be infective. Successful operation on a case of perforation of the gall-bladder following typhoid ulceration is recorded.[12] Owing to preliminary adhesions, perforation may occur into a neighbouring viscus, causing an abscess therein.

[illegible]æ are the result of operation or ulceration. Fistulæ into hollow viscera usually heal spontaneously. Pathological surface fistulæ usually open at the umbilicus. Fistulæ usually require operation. If the ducts are clear an attempt may be made to close the superficial orifice, or separate the gall-bladder by an operation. If the cystic duct is permanently occluded, the gall-bladder must be removed, or cholecystenterostomy performed.

**Intestinal Obstruction from Gall-stones** may occur from early or late peritonitis, volvulus, or from direct obstruction by the stone itself. Large stones may often pass, small stones may cause fatal obstruction. The mortality of obstruction from gall-stones is 50 per cent. With such a high medical mortality, and the frequent impossibility of distinguishing the symptoms from those of an internal hernia, or a band, the question of operation should not be lightly waived or delayed.

**Tumours of the Gall bladder.**—Simple tumours of the gall-bladder may occur from mere distension with the bile, mucus, pus, or concretions. An adipose tumour has been found. The combination of tumour jaundice and cachexia is very suggestive of malignant disease. Gall-bladder tumours have to be distinguished from (1) moveable right kidney, (2) renal or supra-renal capsule tumour, (3) tumour or fæces in bowel, (4) tumour of liver, (5) of pylorus, (6) abnormal projection of liver. Cancer of the gall-bladder is usually secondary to gall-stones or to cancer of adjoining organs. The common form is columnar-celled carcinoma of the mucous glands. Eighty per cent. of cases of cancer of the gall-bladder are associated with gall-stones. It is rarely that the disease is amenable to cholecystectomy.

**Tumours of the Bile-ducts.**—Malignant tumours are the most important, and may be primary or secondary, and may be carcinomatous or sarcomatous (as is the case with the gall-bladder). Probably the relationship with gall-stones is intimate. The diagnosis of new growth of the bile-ducts from gall-stones is practically impossible. Where treatment is available, cholecystenterostomy is far superior to choledochostomy.

**Injuries of the Bile-ducts** must be treated according to the nature of the lesion. If an injury to the common duct be only slight, it may be sutured, but if severe, then cholecystenterostomy is necessary.[13]

**Statistics of Gall-stone Operations.**—Hans Kehr[14] gives the results of over 200 abdominal sections for gall-stones, with operations taking from a quarter of an hour to five hours. The majority were married women who had borne children. In cases where the gall-bladder was not removed, only one patient died out of 127 operations. When the gall-bladder was removed, one in 20 was fatal. In 17 cases the diagnosis proved erroneous, and in 19 more the gall-stones were complicated by more serious disease. Total mortality, 8 per cent. for all operations.

"**The Surgical Significance of Gall-stones**" is the title of a paper by Dr. F. Lange.[15] He advocates an oblique incision parallel to the costal border, and recommends resection of some of the costal cartilages. Attention is directed to the hard enlargement of the head of the pancreas sometimes met with associated with gall-stones.

**Cases.**—A few cases of cholecystenterostomy have been published recently, such as those of Dr. F. H. Peck (Utica),[16] for malignant disease around the ducts; Mr. Binnie (Kansas City), successfully operated on an old lady of 80 years;[17] and Dr. Broca, upon a patient who had already had vaginal hysterectomy performed.[18] Of cases of cholecystectomy the following may be named: Heiderhain's case,[19] in which he removed a gall-bladder, the seat of malignant disease, together with a portion of the liver, by means of Paquelin's cautery;[19] Von Török's case, in which the cystic duct was occluded and injured by gall-stones, but the hepatic and common ducts were patent;[20] Mr. Shettle's, done in two stages;[21] and Dr. David Greig's (Dundee) case, already referred to, in which a portion of the liver was also removed by the aid of ligature.[9]

With regard to the technique of cholecystotomy, it has been recommended at times to sew the parietal peritoneum to the gall-bladder, or even to strip it from the parietes and tuck it down with or without suture (Lange[15]). A case was treated in this way and described by Mr. Charles Morton.[22]

Other cases recently referred to are the following: Several cases with special features, by Drs. Gerster and Lange;[23] original cases by Dr. Marcy (of Boston), who claims to have been the first to plan and carry out the operation of choledochotomy, even before Courvoisier;[24] four cases of cholecystotomy and choledochotomy, with suture and without drainage, all successful;[25] gall-stones complicating malignant disease, cholecystotomy,[26] and impacted gall-stone; Murphy-button anastomosis.[26] choledochotomy with drainage, but without suture,[27] choledocho-lithotomy,[28] and cholecysto-gastrostomy.[29]

A general description of the technique of choledocholithotomy is given by C. Fenger, of Chicago, in the *Annals of Surgery* for December, 1896.

It is a pity the names of gall-bladder operations are so clumsy, but fortunately their meaning is more convenient than their pronunciation.

[11] Brit. Med. Journ., Mar. 13, 1897, and Brit. Med. Journ., Mar. 20, 1897. [12] Lancet, Mar. 2, 1895. [13] Treatment, May 25, 1897. [14] Arch. f. Chirurgie, 1896, vol. 53, p. 362. [15] Johns Hopkins Hospital Bulletin, Feb., 1897. [16] N. Y. Med. Record, April 10, 1897. [17] Annals of Surgery, Nov., 1896. [18] Medical Week, July 17, 1895. [19] Epit. B. M. J., Mar. 6, 1897. [20] Wien. Klin. Woch. [21] Lancet, Nov. 14, 1896. [22] Practitioner, May, 1897. [23] N. Y. Med. Record, April 24, 1897. [24] Annals of Surgery, Jan., 1897. [25] Frederick Page (Newcastle), Lancet, Dec. 5, 1896. [26] Hollowbush, Med. Record, Oct. 10, 1896. [27] Bishop, Med. Chronicle, April, 1897. [28] Johnson (Roosevelt). [29] Ternier, Amer. Journ. Med. Sciences, Jan., 1897.

